# #NicotineAddictionCheck: Puff Bar Culture, Addiction Apathy, and Promotion of E-Cigarettes on TikTok

**DOI:** 10.3390/ijerph19031820

**Published:** 2022-02-05

**Authors:** Makayla Morales, Alexis Fahrion, Shannon Lea Watkins

**Affiliations:** 1 College of Public Health, University of Iowa, Iowa City, IA 52242, USA; makayla-morales@uiowa.edu; 2 Community and Behavioral Health, College of Public Health, University of Iowa, Iowa City, IA 52242, USA; alexis-fahrion@uiowa.edu

**Keywords:** vaping, qualitative analysis, new media, nicotine

## Abstract

This study aimed to classify and delineate types of user-generated content about the disposable e-cigarette Puff Bar shared on the popular video-based social media platform TikTok. We qualitatively analyzed 148 popular TikTok videos collected in July 2020. During an iterative process of data reduction and thematic analysis, we categorized videos by overarching genres and identified emergent themes. Young adults were engaged at all stages of the research process. Together, videos were viewed over 137 million times on TikTok. Seven genres of Puff Bar content emerged: skits and stories, shared vaper experiences, videos to show off, product reviews, product unboxing, promotion of Puff Bar, and crafts. Videos depicted Puff Bar users’ apathy about addiction and a lack of concern of the health effects of e-cigarette use. Additionally, Puff Bar promotion content from underground retailers was extensive and some targeted underage persons. Qualitative analysis of social media content can richly describe emerging online culture and illuminate the motivations of adolescent and young adult e-cigarette use. Social media can facilitate new product adoption; comprehensive e-cigarette regulation and enforcement can counteract these effects by closing loopholes through which new products emerge.

## 1. Introduction

Social media has become a key medium for pro-e-cigarette messaging [1]. On social media, direct advertising from e-cigarette companies, posts from social influencers (i.e., social media users that have credibility and influence in a specific market or brand [2]), and content from peers shape tobacco-related culture and norms and affect personal intentions and use of tobacco products [3,4,5]. This content especially impacts adolescents and young adults in the United States who are both a target market for the e-cigarette industry and frequently use social media; almost all U.S. teenagers and young adults (93%) use at least one social media platform [6], and over half of youth aged 13–17 have reported exposure to some form of tobacco-related content on social media [7].

For adolescents, more time spent on social media is associated with greater intention to use e-cigarettes, and exposure to and engagement with tobacco-related content have been associated with positive attitudes, norm perceptions, and intentions [4]. Among young adults, greater exposure to e-cigarette content on social media was associated with subsequent e-cigarette use and positive perceptions of the effects of e-cigarettes on affect regulation [5,8]. Specifically, one study found that college students who had been exposed to any tobacco product advertising on social media were 33% more likely to be using e-cigarettes at one-year follow up [5]. E-cigarette use by both adolescents and young adults has been linked to an increased risk of acute respiratory issues [9], poor mental health [10], impacted neurological development, and subsequent use of other substances [11,12,13].

E-cigarettes have evolved quickly since their 2007 introduction to the U.S. market. Cigarette lookalikes were swiftly replaced with large, customizable devices with more appealing flavors and then high-tech and discrete “pod vapes” (e.g., JUUL) [14]. The nicotine “buzz”, sleek design, appealing flavors, and concealability of pod vapes encouraged uptake among young people [15,16]. Among middle and high school e-cigarette users, use of emerging disposable e-cigarette devices with similar appearance to pod vapes increased by 400% and 1000%, respectively, between 2019 and 2020. Puff Bar, the most popular disposable e-cigarette, controlled 51% of the disposable e-cigarette market in 2021 [14]. Part of Puff Bar’s initial rise in popularity has been attributed to the exemption of disposable e-cigarettes from an FDA policy action (January, 2020) against flavored cartridge-based e-cigarette products including fruit and mint [17], which enabled Puff Bar to sell a variety of otherwise targeted sweet flavors including “Strawberry”, “Blueberry Ice”, and “Cool Mint” [14,18], and to the removal of cartridge-based flavored e-cigarettes from the market by some e-cigarette companies.

E-cigarette content on social media has evolved to appeal to young people through peer-generated content and references to popular trends. E-cigarette users (i.e., “vapers”), especially young people, prefer social media content about e-cigarettes that reflects their peer group. A study of e-cigarette content on YouTube reported that user-generated videos such as reviews, unboxing, and product demonstrations were more popular than company advertisements [19]. Similarly, e-cigarette posts on Instagram with “activity and product-themed images” received more likes than advertisements [20]. The e-cigarette industry has capitalized on this preference by utilizing peer-driven, trendy content in their social media advertising. Most notably, JUUL’s rapid increase in popularity in 2015 was expedited by its Instagram advertising campaign that featured young models who reflected social trends (e.g., popular social media poses, models wearing fashionable clothing) and used celebrities, social influencers, pop culture references, and hashtags to promote their products [21]. The industry has further capitalized on the appeal of peer-generated content by shifting from direct advertising to promotion via social influencers. Social media has furthermore “democratize[d] mass media” by providing a platform for individuals to share their own content [22] (p. 139).

Given the significant role of social media in the lives of young people, identifying the e-cigarette-related social trends and messages that young people are exposed to in their social media environment is necessary for public health messaging and tobacco regulatory science that is relevant to and effective for adolescents and young adults. Qualitative analysis allows researchers to immerse themselves in the content that young people are exposed to and leverage the full richness of social media text and videos to provide new perspectives. The flexible and open-ended nature of qualitative approaches can produce insights that researchers might not have been looking for, an important feature that can reveal cultural details not identified a priori [23,24].

TikTok is a relatively new, popular social media platform where users create, share, and view short videos. User-generated content includes comedy, education, dances, and news. TikTok was the most downloaded non-gaming app worldwide in 2020 and has nearly 66 million U.S. users [25], 62% of whom are between the ages of 10 and 29 [26]. Similar to other social media platforms, TikTok videos can go viral, meaning that they have high viewership, likes, or shares. TikTok’s novelty to both users and scientific research and its popularity among youth and young adults make it an important platform on which to surveil e-cigarette content.

The objective of this study was to classify the types of user-generated content and messages about Puff Bar on TikTok, describing the emerging culture to support efforts to counteract youth susceptibility to e-cigarette exposure on TikTok and other video-sharing apps. Focusing on widely viewed content about a popular e-cigarette brand on the most popular social media platform allowed us to elucidate the prominent themes in peer-to-peer content at the height of a new e-cigarette trend.

## 2. Materials and Methods

### 2.1. Study Design

We conducted an in-depth qualitative analysis of the most popular Puff Bar videos posted on TikTok in July 2020, interpreting the intent, influence, and messaging of these videos. In qualitative research, researchers are considered sources of creative insight essential to the research process, rather than sources of bias, and add value to the research by leveraging “their theoretical perspective, bank of knowledge, personal experience, methodological repertoire, creativity and imagination” [24]. Knowledge of platform intricacies and cultural knowledge of trends and terms is vital to qualitative interpretation of social media data. The engagement of young adult “cultural insiders” in the study of tobacco products on social media enhances the design and interpretation of the research and ensures that our science is relevant to young people [27].

Our approach, therefore, centered the knowledge, experiences, and creativity of young people. MM, an undergraduate student (age 20), led all stages of project conceptualization, data collection, and analysis; AF, an MPH student (age 22), supported data analysis; MM and AF were thoroughly involved in the interpretation and writing. Both MM and AF are active TikTok users and have been impacted by e-cigarette use in their schools and social circles since they were teenagers. SLW was engaged as a collaborator and mentor at all stages of the research, and we sought feedback from student and faculty colleagues throughout the research process.

### 2.2. Data collection

**Figure 1:**
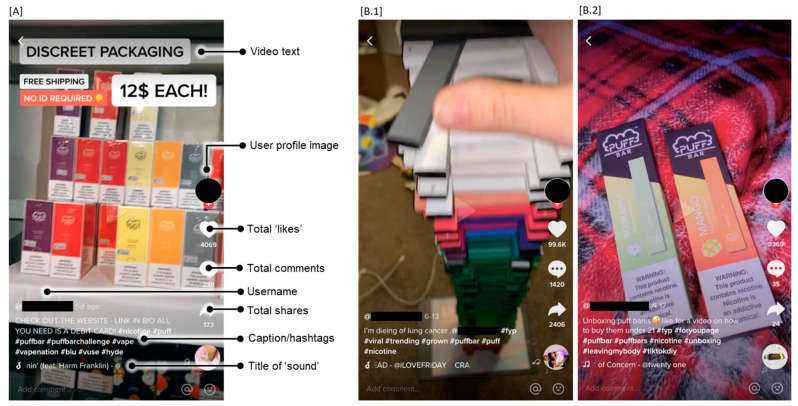
Anatomy and themes of TikTok posts. (**A**) TikTok posts comprise a short video, a caption, hashtags, video text added to the video, comments from other TikTok users, and a pre-recorded audio clip called a sound. Each video also denotes the username and profile image of the content creator and the current number of likes, comments, and shares. (**B**) Together, these features portray the reported genres and themes. (**B.1**) For example, a video of a tower of used Puff Bars, an audio clip repeating “I’m a crackhead, ay”, and the caption “I’m dieing of lung cancer” exemplify the “showing off” genre; the volume of Puff Bars implies high consumption, and the juxtaposition of these features suggests apathy toward the consequences of that consumption. (**B.2**) The “unboxing” genre showed the Puff Bar packaging and device and was used to share information and advertise underground sales, including to underage users.

The primary content in each TikTok post is a short video; at the time of our study, videos could be up to 60 s. Other features include a caption in which the content creator can share brief words to accompany and contextualize the video (e.g., “Unboxing puff barss 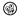 like for a how to buy them under 21”), hashtags to describe the content and themes of the video (e.g., #noaddictionhereboys), video text that provides written commentary during a video (e.g., “Part 2 of me delivering puff bars to 18+ during quarantine”), comments from other TikTok users, and a pre-recorded audio clip called a sound. Each video also denotes the username of the content creator (Figure 1A). TikTok’s “For You Page” curates individualized content for each user with an algorithm based on the user’s previous engagement with TikTok content through likes, comments, and shares [28]. Creators may attempt to boost their content’s popularity by adding hashtags that are trending or relevant to the topic of their video and using popular sounds. In this study, we used the video, caption, hashtags, video text, and sound to interpret the meaning of each post (Figure 1B).

To identify popular content, we searched for videos using the five most popular Puff Bar-related hashtags with over one million views: #PuffBar, #Puff, #PuffBars, #PuffBarChallenge, #PuffPlus, which ranged from 146.4 million views (#PuffBar) to 7.9 million views (#PuffPlus). For each search, we selected the five most popular videos, ignoring duplicates, as determined by TikTok’s algorithm using views, likes, comments, and popularity of hashtags. A video was included if it was in English, contained at least one relevant hashtag, was posted on or after 1 January 2020, and had an engagement rate of at least 0.20 (calculated as the sum of likes, comments, and shares divided by the number of views), a rate that signals high engagement (https://theinfluencermarketingfactory.com/ accessed on 30 January 2022). We included videos with a Puff Bar hashtag, regardless of whether the content actually related to Puff Bar, a decision informed by our tacit knowledge of hashtag use on TikTok, where it is common practice for creators to use unrelated but trending hashtags to increase the reach of their posts. We conducted the full set of searches six times between 1 July 2020 to 12 July 2020, varying the time of day we searched in order to produce a wider variety of content. We downloaded each video and saved a video still to document the caption, hashtags, and username. We screened 864 videos, and our final dataset included 148 videos.

### 2.3. Data Analysis

We analyzed TikTok videos qualitatively to identify emergent themes related to Puff Bar use and popularity. Authors MM and SLW conducted an iterative process that involved viewing videos, taking notes, and discussing emerging impressions to develop an initial codebook. After testing and refinement, our final codebook included 19 codes related to the video type or purpose (e.g., “unboxing”, “social commentary”) and the content discussed (e.g., “addiction”, “obtaining a Puff Bar”) and three categories for data management: “miscellaneous”, “notable posts”, and “unrelated to Puff Bar”.

MM led coding and analysis. First, we coded each video with all relevant codes (Microsoft Excel). For each code, we then watched and summarized each video, wrote analytic memos to document emerging ideas, and identified patterns using these summaries and memos. We then organized all of our analytic notes across codes using the collaborative online whiteboarding app Miro, identifying, labeling, and reaching consensus on emerging themes. We returned to original videos to confirm findings. This iterative process produced the final video genres and themes described below.

This study was determined not to be human subjects research by the University of Iowa Institutional Review Board. Ethics of social media research continue to evolve and concerns have been raised about whether social media users conceptualize their posts as “public” [29]. We believe that our research presents limited risk to content creators because the videos in this study are posted in a public forum (viewable even without a TikTok account) and are not only widely viewed but are designed to engage a large audience. To maintain the anonymity of the creator, we do not report any identifying information and use brief descriptions of content. Below, we use the generic “Puff Bar” to refer to several specific products featured in these videos.

## 3. Results

The 148 videos in our dataset had an average engagement rate of 23.8; videos had a median of 44,900 likes, 324 shares, 230 comments, and over 200,000 views. Collectively, the videos had been shared approximately 370 thousand times and viewed over 137 million times. Videos were shared by individual Puff Bar users, vape shop and other retail workers, and individuals who sold Puff Bar outside of retail settings (i.e., “underground”). Although we were not able to confirm demographic characteristics of content creators from observation alone, the videos largely featured creators who appeared to be teenagers or young adults (consistent with evidence that 60% of TikTok users at the time were aged 16–24; https://wallaroomedia.com/blog/social-media/tiktok-statistics/ accessed on 30 January 2022) and White.

Findings emerged in two categories. A first set of findings classified the types of videos into “genres” based on content and style. The second delineated Puff Bar perceptions and behaviors across video genres. Findings in both categories emphasized the wide popularity of both Puff Bar content on TikTok and Puff Bar use.

### 3.1. Genres of TikTok Video Content

Emergent coding and thematic analysis revealed seven “genres” of related video content: skits and stories, shared vaper experiences, product reviews, product unboxing, promotion of Puff Bar, videos to show off, and crafts. A small group of videos were unrelated to Puff Bar.

Many videos featured skits which had organized and coordinated efforts to make a joke or statement related to Puff Bar. For example, a subset of “skit” videos produced by vape shop or convenience store workers made statements or presented tropes about Puff Bar users based on their experiences, but not necessarily representing a real event. For example, a skit from a convenience store employee highlighted an apparent contradiction of disposable vape use: in the skit, a Puff Bar user complained about spending too much money on Puff Bars each week. The employee recommended a $60 reusable nicotine device to save the customer money in the long run, but the customer declined, claiming that $60 was too much money to spend upfront. The customer simultaneously spent that same amount on Puff Bars. Another subset of videos featured storytelling by creators recounting Puff Bar-related events from a first-person perspective. For example, one creator recounted an experience running into the mother of a young buyer while they were delivering an order of Puff Bars. The creator described leaving the Puff Bars in the mailbox, noting how conspicuous it was and their embarrassment.

A related set of videos depicted a variety of shared vaper experiences, or experiences considered relatable among Puff Bar users. For example, creators voiced the hardships of cessation and complained about so-called “nic fiends” who constantly asked to “take a hit” from their friend’s vape. Vape shop employees shared information about Puff Bar and Puff Bar culture through skits, storytelling, and videos about shared vaper experiences. These employees were a significant information source and received many questions from their audience about product legitimacy, brand and flavor recommendations, and other topics.

Videos commonly offered product review and/or depicted product unboxing. In product reviews, the content creator shared opinions and/or facts about Puff Bar, sometimes offering commentary as they opened and used a new Puff Bar. Unboxing videos depicted the act of opening a Puff Bar either in a clip of a person opening the product or a set of clips that displayed the product at each stage of unboxing (i.e., the box, the inner packaging, and the actual device). Unboxing videos typically highlighted the packaging, flavors, and aesthetics; they also sometimes included a product review.

The largest genre from our dataset was promotion of Puff Bars, which included videos from multiple sources, including TikTok users who sold Puff Bars outside of a retail setting (i.e., Puff Bar “dealers”), “Puff Bar influencers” who made a percentage of the profit of Puff Bars from third-party websites when customers used the influencer’s promotional code, vape shop workers, and individuals promoting Puff Bar without financial incentive. Dealers were easily distinguished from fans of Puff Bar by their unique language. Some stated explicitly and others hinted that they sold Puff Bars. They also encouraged viewers to add them on Snapchat or follow them on Instagram, and often promoted an Instagram handle that referenced that they were a Puff Bar dealer. Videos from dealers were often unboxing videos, reviews, or displays showing off their volume of product.

The final core genre included videos to show off. Creators displayed the Puff Bars they had collected over time, often several dozen; in a unique subgroup of these videos, creators lined up their collection on a table and pushed them off onto the ground or into a bin. These specific videos were often accompanied by an audio that included a voiceover saying “nicotine addiction check” followed by coughing noises over a drumbeat. Addiction was also mentioned in captions or text (e.g., “when you took a break & finally hit it 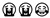
”). These collections and the accompanying commentary implied significant use and addiction by the creators.

Several videos depicted construction of crafts with old Puff Bars, including a rolling tray for marijuana joints and an ashtray. Six videos made no reference to Puff Bar except in the hashtag, which supported our supposition that the popular hashtags might be used to increase visibility of unrelated content. These six videos were not included in further analysis.

### 3.2. Perceptions and Behaviors

Three primary themes related to perceptions and behaviors emerged across genres: the minimization of addiction and health consequences from Puff Bar use, a seemingly contradictory concern for the legitimacy of Puff Bar devices, and widespread underage use and sale of Puff Bar. A fourth theme revealed how creators used the features and nature of the TikTok platform to communicate.

#### 3.2.1. Addiction Apathy

Addiction among young Puff Bar users is indirectly evidenced by an entire “showing off” genre of videos depicting large collections of old Puff Bars and videos, including skits, that depicted the lengths users went to vape Puff Bar. However, we found that many Puff Bar creators were apathetic towards addiction, and even vaped for the side effects, including appetite suppression and eased bathroom use. Users displayed their apathy in videos that made light of the addictive effects they were experiencing, for example by juxtaposing a comment, hashtag, or audio that mentioned addiction with a video that displayed Puff Bar use or dependence with no concern. One creator described how they only drink water, eat rice cakes, and vape their Puff Bar, captioning their video “self care all 2020”. In another video, the creator found and used a discarded Puff Bar at the beach. The creator’s disregard for health is emphasized by an accompanying upbeat hip hop sound with lyrics “I’m a crackhead, ay, I’m a crackhead” and is especially significant given they recorded this video during the COVID-19 pandemic in 2020.

Similarly, other health-related commentary was largely made humorously, although health messages were less common than addiction messages. For example, a lighthearted video of approximately 200 Puff Bars arranged by color in a tower is captioned, “I’m dieing of lung cancer” and enhanced with the same upbeat sound “I’m a crackhead, ay” (Figure 1B).

#### 3.2.2. Legitimacy Concerns (and Lack Thereof)

In contrast, Puff Bar users on TikTok seemed to be concerned about the legitimacy of Puff Bars. For example, a vape shop employee reported receiving hundreds of comments a day asking how to spot a fake Puff Bar, provided a step-by-step tutorial on how to use Puff Bar’s online product authentication system, and showed viewers how to find packaging discrepancies between legitimate and illegitimate products. Many unboxing videos emphasized the QR code by explicitly mentioning or zooming in and out on the code, which suggests that legitimacy was a concern of their viewers. Still, several videos about legitimacy reflected the concern’s limited impact on behavior. For example, during an unboxing video, one creator asserted that they would vape the strawberry watermelon Puff Bar they just discovered was fake anyway because “that’s the kind of person I am”.

#### 3.2.3. Underage Promotion

Puff Bar videos included information for minors. Both promotional and informational videos described where to purchase under the age of 21 and advertised discreet packaging that would help minors hide their Puff Bar use from their guardians; several underground Puff Bar dealers used the hashtag #forthelow in order to share with their underage viewers that this creator would be able to sell and deliver Puff Bars in a discreet, under-the-table manner. Several videos from an influential vape shop worker referenced selling nicotine products to minors, one of which joked about how they even sell e-cigarettes to people with obviously fake IDs. However, some content did emphasize age restrictions. For example, one vape-shop employee stated, “FYI we card everyone! Vaping is for 21+ and up in the state of California”.

#### 3.2.4. Enhancing Communication with TikTok Tools

We found evidence that creators utilized TikTok features to enhance their message and/or to go viral. Creators added sounds, hashtags, and video effects to emphasize their point; for example, the bling effect added sparkles to unboxing videos to increase the appeal of the product. Some creators appealed to the “viral” nature of TikTok in their messages, for example, one Puff Bar user told viewers if they “blow this video up” the creator would quit Puff Bar. It is likely that the six unrelated videos in our dataset, for example a video about styling a wig on a mannequin, used a Puff Bar hashtag in order to increase their reach.

## 4. Discussion

Our qualitative thematic analysis of popular Puff Bar videos on TikTok illuminated a “Puff Bar culture” among adolescents and young adults, evidenced by shared jokes and commentary about Puff Bar, the repetition of content such as the “nicotine addiction check” meme and the unboxing format, and similar utilization of TikTok features across creators. We identified content genres that reflected user experiences and beliefs (skits and stories, shared vaper experiences, videos to show off, crafts) and promoted Puff Bar (product reviews, product unboxing, promotion of Puff Bar), especially to underage users. Creators employ TikTok features, jokes, skits, and commonalities between Puff Bar users to promote and discuss Puff Bar.

Our findings are consistent with the themes reported in an Industry Watch analysis of 10 popular Puff Bar videos on TikTok: sale or promotional content, nicotine and addiction-related content, youth-related content, and flavors. Authors of that study also noted that videos often contained audio features, hashtags, and jokes that seemed to appeal to a larger culture of Puff Bar use and discussion on the app [30]. Additionally, a systematic analysis of vaping on TikTok reported some comparable categories, including “comedy and joke” and “nicotine and addiction”. Our analysis adds rich description and interpretation by probing the videos within these categories for deeper meaning, describing dominant shared narratives, and identifying ways in which platform features perpetuate these messages [31].

TikTok became a “place” for conversation where creators exchanged information about Puff Bar products, flavors, and experiences. Other studies have described similar e-cigarette communities on other social media platforms [32,33]. However, a study on Twitter reported that 97% of disposable e-cigarette-related tweets from March to September 2020 were from commercial sources rather than users [18]. While we did analyze only the most popular videos during the study period (which are more likely to be user-generated), we suspect this methodological difference does not sufficiently explain the difference in findings. The video-based nature of TikTok lends itself to “storytelling” between users, which might be particularly relevant for portraying the visual and tactile experience of using Puff Bars, and to the creation and popularization of memes that other users participate in and share. Additionally, commercial content might not have yet become established on TikTok as it had on older platforms, and young people using Puff Bar might not share content about their substance use on Twitter (e.g., because they do not use the platform or would face social repercussions). We encourage further investigation of image-rich content (e.g., on Instagram, TikTok) that might reveal unique facets of online tobacco culture.

Although we cannot verify the age of the creators or buyers, we found substantial evidence of promotion of underground and underage sales, consistent with previous findings that other platforms also facilitate access to both information and e-cigarette products for adolescents [34]. In addition to having increased access to dealers, adolescents may be encouraged by adult users to vape Puff Bars through content that makes it look like access when underage is simple, glamorizes vaping by creating a “Puff Bar aesthetic”, and minimizes the effects of addiction.

In our data, TikTok features (e.g., audio clips) were used to highlight packaging, facilitate jokes, increase appeal, and increase interaction and viewership. The appealing imagery used on TikTok is consistent with content on other social media platforms that targets youth and young adults. For example, e-cigarette promotions on Instagram were found to be colorful and visually appealing [35], and over 50% of JUUL-related content collected from March to May 2018 was found to reference youth lifestyle and culture [36]. TikTok content might glamorize Puff Bar use and reinforce positive beliefs about e-cigarettes, consistent with previous findings that exposure to e-cigarette content on social media can increase positive perceptions of use [8]. TikTok users are essentially doing the marketing work for the e-cigarette industry, as well as promoting underage and underground sales.

TikTok’s unique format might additionally reinforce Puff Bar’s appeal and the problematic messages that we have described. The agency that social media users have to filter content can create a “bubble” that reinforces pro-tobacco norms. While social media sites all use software designed to sort content in a way that peaks and maintains interest (i.e., an algorithm [37]), TikTok’s algorithm is unique. Other social media applications simulate real-world social interactions with a group of friends or followers, and they prioritize content from that friend-group in a user’s feed [38]. Alternatively, TikTok’s “For You Page” presents users with a digital replication of their own identity that reinforces their interests [38]. TikTok’s algorithm might be particularly effective at producing an information bubble, filtering out anti-e-cigarette messaging and potentially saturating susceptible young people with pro-tobacco content. Research on anorexia in young girls demonstrates a similar phenomenon. For example, in one case report, a 14-year-old girl’s “For You Page” was saturated with videos by creators who appeared to be competing with one another over who was more affected by anorexia [39].

### 4.1. Implications for Research and Practice

Our findings contribute to the growing body of work on pro-tobacco content on social media by providing novel insights on the presentation and communication of a new device on a highly popular social media platform. Our work re-emphasizes the importance of social media in the socio-ecological context of youth e-cigarette use as a source of informal marketing, social engagement, and product access, and the need for innovative social media interventions (e.g., Truth’s 2020 “Ready to Ditch JUUL” campaign kicked off with a TikTok challenge [40]). Regular surveillance that leverages the richness, subtleties, and adaptability of qualitative analysis can identify emerging tobacco-related narratives on social media and inform effective prevention and cessation interventions. Furthermore, our study offers one example of how to approach a qualitative analysis of social media content, offering several strengths that can be integrated into future work [24]. The engagement of young researchers enhanced scientific rigor in both research design and interpretation of content by employing their tacit knowledge of social media platforms and insider perspective as members of our target population. We also provide an example of methods to analyze image-rich social media data, a growing research domain.

Mirroring the trajectory of JUUL, Puff Bar offered a new, convenient, and appealing product without FDA authorization, and quickly established a young market. Popularity skyrocketed despite indications that Puff Bar products might have been unsafe (e.g., inconsistent packaging, leaking devices [41]). Reactive e-cigarette regulation provides windows for new products such as Puff Bar to arise, which might undermine the efficacy of tobacco control targeted at reducing nicotine addiction and other e-cigarette risks among young people [42,43]. In March 2021, Puff Bar began using nicotine that was not derived from tobacco in their products, testing another potential loophole in FDA’s regulation and enforcement [44]. Comprehensive e-cigarette regulation and enforcement are imperative to prevent new trends from emerging through regulatory gaps.

We found a dominant narrative that nicotine addiction and long-term health consequences from using Puff Bar were not concerns, consistent with other reports of addiction apathy among young people [45,46]. Previous findings also suggest that young people want straight-forward health information about e-cigarettes [46]. Public health messages designed to increase salience of addiction and health risks might focus on more relevant concerns such as legitimacy of the devices and mental health and deliver clear and level information (e.g., https://www.thetruth.com/hot-topic/vaping-mental-health accessed on 30 January 2022). From our data, we were not able to explain why users were concerned about product legitimacy. Our finding that health concerns are low might be attenuated if legitimacy concerns were about product quality (e.g., poor nicotine delivery) and not about harm. Moreover, the QR code verification process created an “air of legitimacy” for “true” Puff Bars even though the producer of those Puff Bars was unknown [41]. This focus on legitimacy generated a narrative that any flawed device was fake [41] and so Puff Bar was seemingly protected from scrutiny. Anti-industry messaging has been found to be appropriate for young smokers [47,48]; messages that engender anti-e-cigarette industry sentiment might have similar salience [49,50].

TikTok’s Community Guidelines state “We do not allow the depiction, promotion, or trade of drugs or other controlled substances. The trade of tobacco and alcohol products is also prohibited on the platform” [51,52]. TikTok provides content warnings on many videos containing alcohol, removes videos, and bans policy violators from posting further content. Despite having the same prohibitions for both alcohol and tobacco, not a single video in our data contained a content warning (and had not been taken down at the time of data collection). Facebook and Instagram have taken steps to reduce underage exposure by prohibiting influencers from posting branded e-cigarette content [53]; TikTok could apply its procedures for alcohol content to tobacco content.

Advertising regulations must consider the connections across media platforms, and again close loopholes that perpetuate tobacco marketing to young people. A recent TV ad for VUSE (RJ Reynolds) closely referenced the “#NicotineAddictionCheck” meme we described above, mimicking the genre’s footage of devices being pushed off of a table [54]. Not only is this copy concerning because it leverages a popular user-generated meme and, therefore, is likely appealing to youth, but by doing so, it likely perpetuates the related cultural narratives of addiction apathy and performance that we describe in this genre.

### 4.2. Limitations

Although we conducted a thorough analysis of the videos in our dataset, our inability to follow up with content creators about their intent limits our interpretation. Social media content is performative and user-generated content may not reflect actual beliefs and behaviors; there is consistent evidence of a gap between reported and actual behaviors [55]. However, the aims of this study were to identify dominant content and messages in the social ecological environment of young people, and our findings should be interpreted with that in mind. We also had limited ability to describe the demographic characteristics of the users in our sample, making it difficult to illuminate any differences in messaging across groups. We hope that our findings can provide a foundation for future research on tobacco-related disparities on TikTok [56]. Our team, a group of female researchers living in the United States, might have missed or misinterpreted content based on the limitations of our lived experiences.

## 5. Conclusions

Our qualitative work contributes an “insider” perspective of user-generated Puff Bar content to the body of literature describing e-cigarette social media content in an evolving landscape. The young adult researchers involved in each step of this study were able to provide a unique perspective on the app and were able to help interpret the different trends, filters, and sounds utilized by creators, allowing for more relevant data interpretation. Understanding online e-cigarette culture is imperative to generating effective anti-tobacco content on TikTok and other platforms. We describe a Puff Bar culture based on common sounds, features, genres, jokes, and product discussions. We demonstrate that much of this content references or targets young users, which is especially concerning because many young users displayed apathy toward the health implications of addiction and because the app’s algorithm might reinforce negative health behaviors. Our analysis provides essential background for effective counter-messaging and highlights the need for stronger social media regulation and tobacco regulatory enforcement that protects youth from overexposure to pro-tobacco messaging online.

## Data Availability

The data presented in this study are available upon request from the corresponding author. The data are not publicly available due to potential for identification of content creators.
